# The AI trilemma: Saving the planet without ruining our jobs

**DOI:** 10.3389/frai.2022.886561

**Published:** 2022-10-19

**Authors:** Ekkehard Ernst

**Affiliations:** International Labour Organization, Department of Research, Geneva, Switzerland

**Keywords:** sustainability, artificial intelligence, inequality, productivity, jobs

## Abstract

Digitalization and artificial intelligence increasingly affect the world of work. Rising risk of massive job losses have sparked technological fears. Limited income and productivity gains concentrated among a few tech companies are fueling inequalities. In addition, the increasing ecological footprint of digital technologies has become the focus of much discussion. This creates a trilemma of rising inequality, low productivity growth and high ecological costs brought by technological progress. How can this trilemma be resolved? Which digital applications should be promoted specifically? And what should policymakers do to address this trilemma? This contribution shows that policymakers should create suitable conditions to fully exploit the potential in the area of network applications (transport, information exchange, supply, provisioning) in order to reap maximum societal benefits that can be widely shared. This requires shifting incentives away from current uses toward those that can, at least partially, address the trilemma. The contribution analyses the scope and limits of current policy instruments in this regard and discusses alternative approaches that are more aligned with the properties of the emerging technological paradigm underlying the digital economy. In particular, it discusses the possibility of institutional innovations required to address the socio-economic challenges resulting from the technological innovations brought about by artificial intelligence.

## 1. Introduction

The past decade has seen an explosion of applications powered by artificial intelligence (AI). With the ubiquity of large, unstructured databases (“Big data”) and a rapid fall in computing costs over the past four decades, AI applications using non-linear statistical and machine learning methods have gained renewed prominence after falling out of favor for long periods since the inception of the field of AI properly speaking. This has triggered both fears about a robo-apocalypse with machines dominating the world as well as enthusiastic techno-scenarios where humanity can solve most of its current global challenges, be they related to climate change, poverty or diseases (Brynjolfsson and McAfee, 2014; Frey and Osborne, 2017; Frey, 2019; Ford, 2021). Yet, none of these scenarios seem to materialize right now. Rather, we see specific challenges arising from the wide-spread use of AI, in particular when it comes to the use of social media. Also, the rising ecological footprint of digital tools—and specifically AI-powered applications—notably as regards cryptocurrencies^1^ and foundation models^2^, has raised concerns about the sustainability of these developments (Robbins and van Wynsberghe, 2022). At the same time, enhancements to our way of life have been equally limited, mostly concentrated around improvements in digital navigation or the rapid rise in online shopping and delivery. At the back of these rather limited effects looms a more concerning trend: the rise in economic power of a few dominant technological companies that increasingly seems to add to inequalities already prevalent before the rise in AI.

By now, all three challenges resulting from the rise of AI are well documented, whether they concern limited productivity gains (Gordon, 2021), worsening inequalities (Bessen, 2020) or rising ecological costs (van Wynsberghe, 2021). This paper argues that these three challenges are interrelated and need to be understood as resulting from an “AI trilemma:” Following its current path the technological paradigm taken by AI will worsen its ecological footprint and deepen economic inequalities without delivering better living standards for all. Using the concept of a technological paradigm as developed by Dosi (1982) and Nightingale et al. (2008), I will argue that at the heart of this trilemma lies a particular way of how this technology develops, related to both technical and economic aspects of its current paradigm. I will also argue that these developments are not inevitable as specific policy interventions and institutional changes can modify this paradigm in such a way as to deliver positive contributions to our way of life without worsening or even with improving on its ecological and social costs to become a truly sustainable paradigm. This point is similar to the one raised by Acemoglu (2022) in as much as the unfettered technological development under the current paradigm is unlikely to deliver the benefits expected from AI; in contrast, I argue that identifying a direction of technological change that delivers these benefits requires to understand the inherent trade-offs between inequality, ecological costs and productivity growth that comes with the current paradigm.

**Figure 1:**
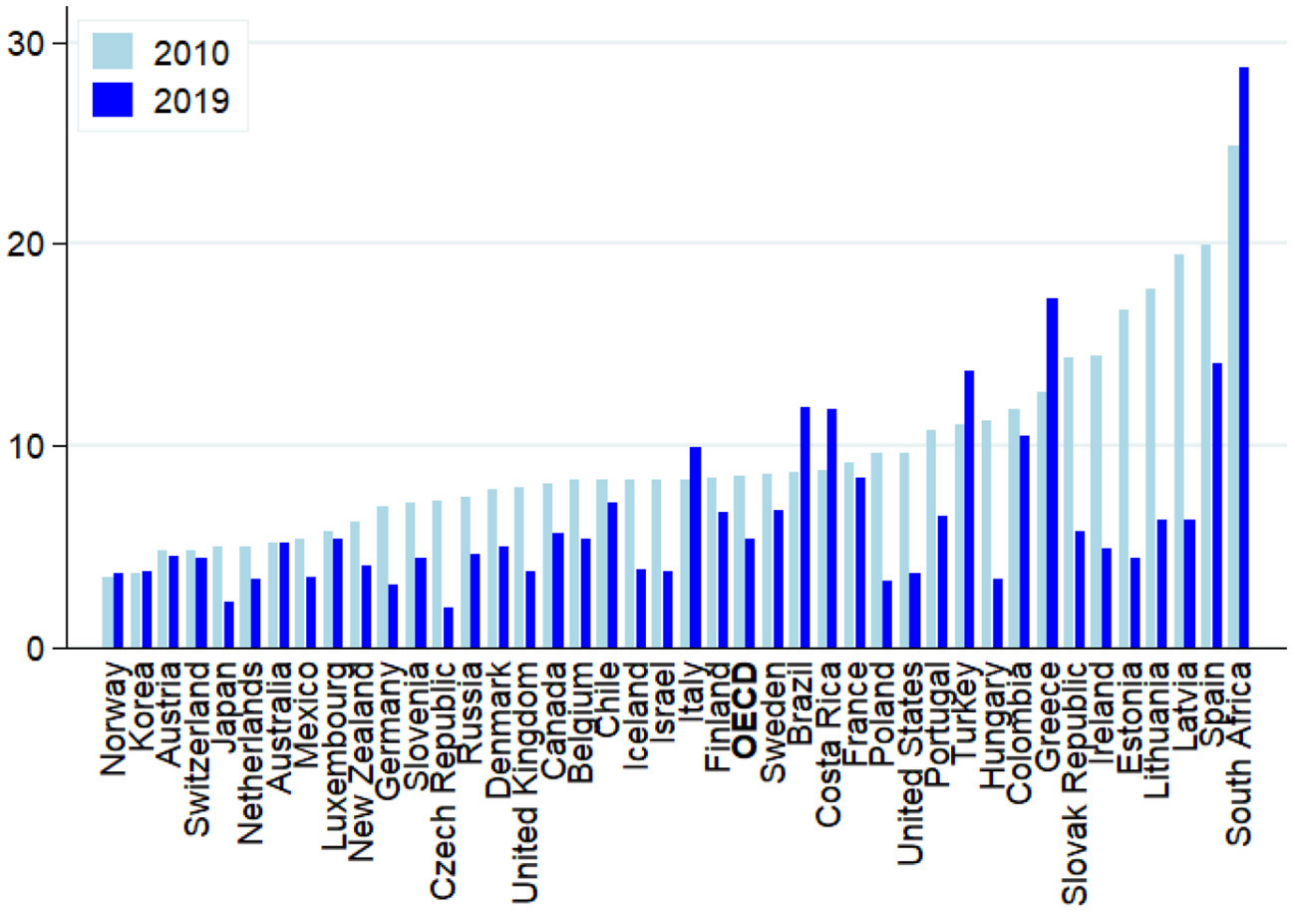
Evolution of unemployment: OECD and selected G20 countries (2010 vs. 2019; in per cent of total labor force). Source: OECD, Stats Portal.

Many researchers and observers focus their analyses of AI on its applications in the world of work, which initially rose fears of wide-spread technological unemployment (Frey and Osborne, 2017; Balliester and Elsheikhi, 2018; Frey, 2019). Whether autonomous taxis, fully automated logistics centers, the Robo-Hotel concierge Pepper or the Bar Tender Tipsy Robot; in more and more areas machines seem to be able to replace us. This is especially true in those areas where we ourselves have been convinced of being irreplaceable: In artistic or intellectual activities (Muro et al., 2019). Calls for a universal basic income or some other unconditional forms of government transfers abound in order to secure all those masses of employees falling out of work and providing them some minimum way of life. In the meantime, however, it seems that (technological) unemployment should be the least of our concerns with these new digital technologies, at least in advanced economies (Carbonero et al., 2018). Indeed, if anything, unemployment has declined in OECD countries during the past decade up until the outbreak of the Covid-19 pandemic (see Figure 1).

**Figure 2:**
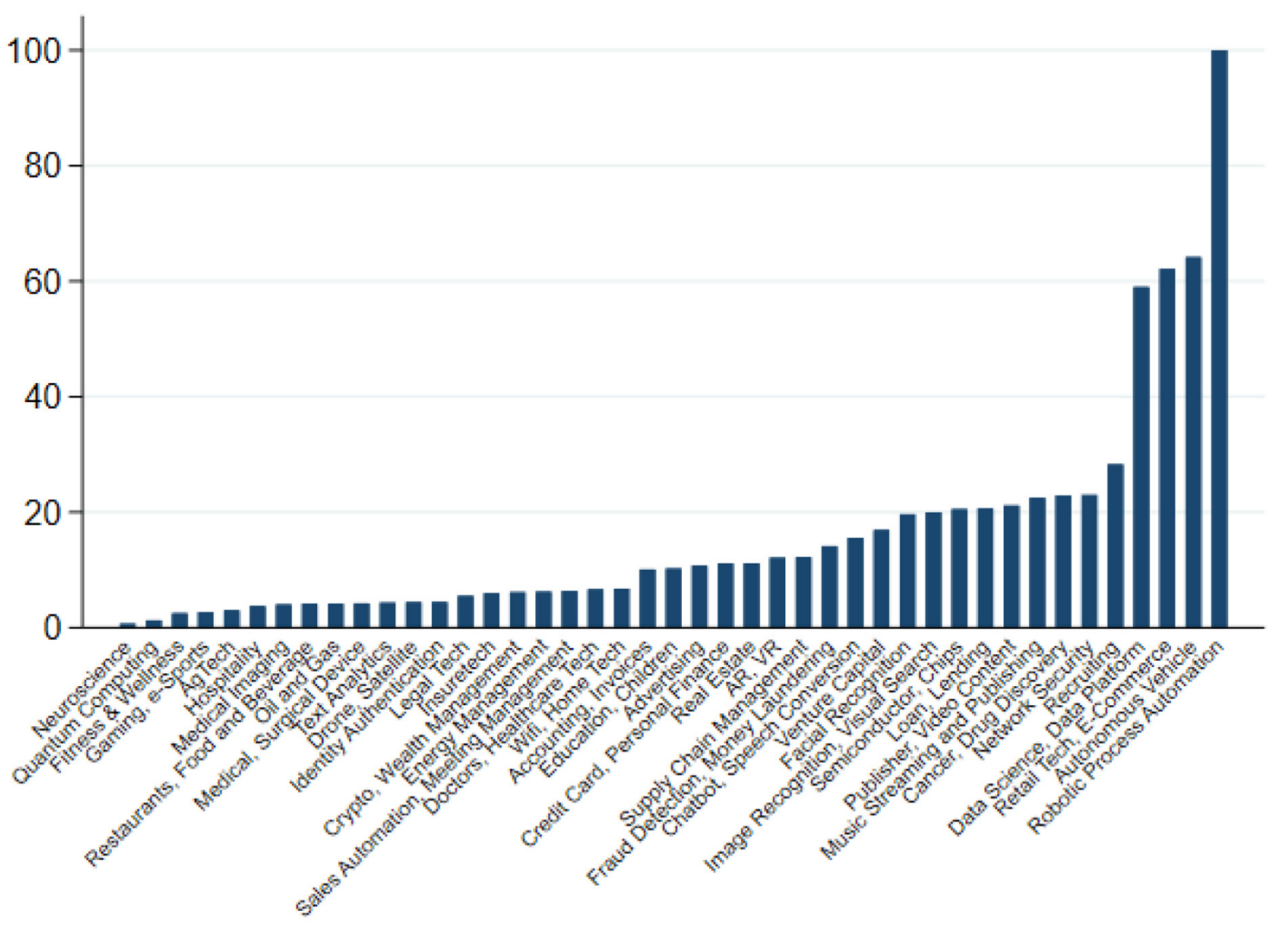
Main areas of AI development (2010–2020, cumulative investment in US$, global; Robotic Process Automation = 100). The chart depicts the cumulative, global investment in US$ over the period 2010 to 2020 in various AI-applications. Investments have been scaled such that total investment in Robotic Process Automation = 100. Source: Ernst and Mishra (2021) based on the Stanford AI Vibrancy index.

Part of the reason why AI-powered applications have so far not led to a job-less future relates to the vary narrow range of applications that are currently being developed by industry (Ernst and Mishra, 2021), affecting only a small percentage of the workforce. Indeed, over the past decade most applications have been centered around business process robotisation, autonomous driving, e-commerce and digital platforms, which together accounted for more than 40 per cent of all applications developed between 2010 and 2020 (see Figure 2). In particular, business process robotisation—such as applications in accounting and compliance—seem to have been developed partly as a reaction to rising compliance cost and regulatory overhead, rather than to substitute employment. Some researchers have even highlighted that many of these applications are likely to prove labor augmenting rather than replacing, possibly leading to job enrichment, which, in principle, should allow workers to command higher incomes and firms to enjoy higher productivity (Fossen and Sorgner, 2019).

**Figure 3:**
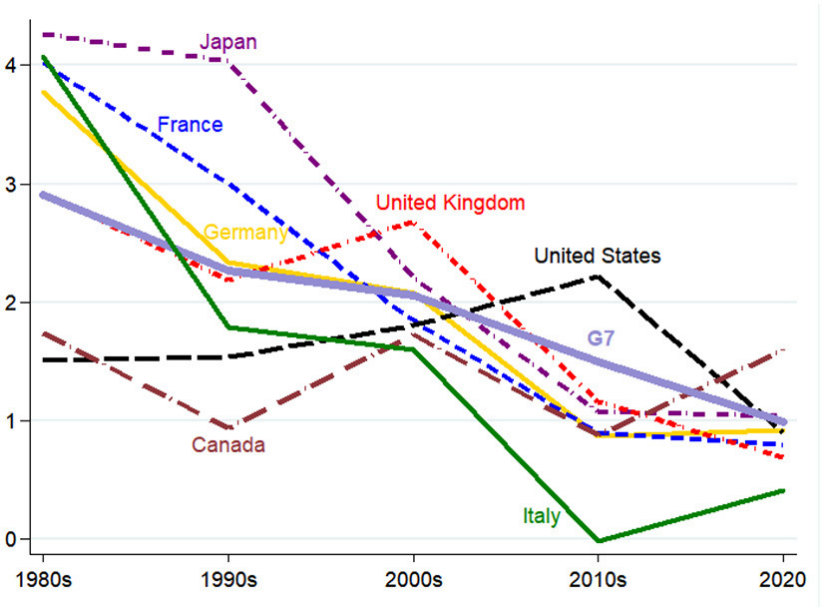
Hourly labor productivity growth (in %, decade averages, 1980-2020, G7 countries). The chart depicts the average hourly labor productivity growth between 1980 and 2020 for G7 countries. Decade averages only available for 1980–1989; 1990–1999; 2000–2009; 2010–2019. Last observation is for 2020 and might be biased due to the effect of the COVID-19 pandemic on national statistics. Source: OECD, Statistics Portal, available at: https://stats.oecd.org/.

Yet, these more positive conclusions also do not seem to have materialized. Productivity growth has continued its secular decline over the 2010s (Ernst et al., 2019) and does not seem to have accelerated with the onset of the recovery as we are gradually moving out of the pandemic. Despite much touted benefits from working-from-home and the further growth in e-commerce, apparent hourly labor productivity growth in the OECD has not increased (see Figure 3), with the possible exception of the United States that saw a gradual increase since the mid-1990s, albeit well below levels achieved in decades prior to the second oil shock in the early 1980s.

Meanwhile the rising ecological cost of developing and using AI has become an important concern. This has become most visible in the area of cryptocurrencies where the particular security concept behind Bitcoin, for instance, has led to an explosion in the use of electricity, up to the point that several countries have restricted or outright banned its use (e.g., China, Kosovo). Other areas of the digital economy have also experienced increasing constraints. Some large digital companies have started experimenting placing its cloud computing servers in deep sea water for cooling. Large-scale neural networks such as the natural language processing network GPT-3, currently one of the largest and most powerful tools in this area, is reported to cost US$ 12 million on a single training run, making it very costly to correct training errors (for instance due to biased data) and effectively preclude a more wide-spread application of this tool, especially by smaller companies (OECD, 2021). What is more, as these tools become more complex and presumably more precise, their economic and energetic costs explode and do not scale up linearly (Thompson et al., 2020). In the meantime, a call for “Green AI” or sustainable AI has emerged, focusing on how to lower the carbon-footprint of these tools and ensure their (low-cost) accessibility of a large range of researchers and users (Robbins and van Wynsberghe, 2022). Various possible technological improvements have been suggested but, so far, none of them seems promising enough to contribute significantly to a solution as we will discuss in more detail below. Presumably, the rise in renewables in the energy mix would bring down the carbon footprint of AI but only to the extent that its use does not continue the exponential rise observed over the past decade, which seems unlikely.

Interestingly, those areas where AI neither replaces nor (directly) complements work have not received much attention. In economic terms, new technologies can affect productivity at three levels: labor, capital or total factor productivity. The latter typically refers to technologies that help combine both production factors in more efficient ways, for instance through re-organization of work processes. More broadly, technologies to manage networks more efficiently, for example in transport and logistics, in electricity and waste management or in information exchange, are prime candidates for improvements in total factor productivity (UN DESA, 2018). Modern urban traffic control systems can use flexible traffic management to direct individual and public transport in such a way that the traffic volume is managed optimally and efficiently. AI will also become increasingly important in the area of electricity network control, especially where more and different energy sources (e.g., renewables) have to be connected as economies are transiting toward sustainable energy supply. Similarly, as economies are trying to reduce their overall ecological burden, waste management will become more important together with an increasing role played by the circular economy. Such (complex) supply chains remain beyond the purview of human intervention and require high-speed control by machines.

So far, however, none of these applications seem to play an important role in the discussion among economists and social scientists about how transformative this technology potentially can be. As I will argue below, this has to do with the particular way the technology business operates and requires a conscious effort to redirect (partly) our efforts in developing innovations in this area. I will start with some methodological considerations before presenting the AI trilemma in a nutshell, highlighting the key mechanisms underlying it. I will then delve into its three main components: lack of productivity growth, rising inequality and market concentration, and a worsening ecological footprint. In Section 4, I demonstrate several areas in which technological progress in the digital work can indeed contribute to address the AI trilemma and present some policy proposals on how to instigate such a change. A final section concludes.

## 2. The AI trilemma in a nutshell: A technological paradigm

### 2.1. Technological paradigms

Underlying the understanding of the AI trilemma is the concept of a technological paradigm as a socio-technological interaction between technological capabilities, economic conditions and social structures that determine the future development of the productive forces of an economy (Dosi, 1982; Nightingale et al., 2008). A technology here refers to a set of combinations between labor, capital and ideas to produce a certain economic output. At its most basic level, technological development then can be either autonomously driven by scientific progress (“ideas”)—the scientific supply push paradigm—or determined by economic conditions under which firms operate on both labor and capital markets—the demand pull paradigm. As such, the concept of a technological paradigm expands on Kuhn's scientific paradigms as one that applies more broadly even outside academic communities.

As highlighted by Dosi (1982), the two ideal forms do not uniquely reflect the dynamics of technological progress, which will inevitably navigate between the available scientific knowledge of any particular era and the specific socio-economic conditions under which firms operate. One shall add to this that either of the two forces will be influenced by institutional and regulatory conditions, such as laws and regulations governing intellectual property rights, tax regimes or government subsidies for R&D, among others.

It is against this concept of technological paradigms that the AI trilemma will be developed in this paper: I will explore how the current scientific and technological development of digital tools in general and AI in particular interacts with the institutional and regulatory regime on labor and capital markets. I will then analyse the specific socio-economic outcomes this interaction produces to show that certain undesirable properties cannot be overcome within this prevailing technological paradigm. As such the AI trilemma is not a logical impossibility to achieve more desirable outcomes with the currently available technologies but rather a contextual trilemma that can be overcome with the right institutional and regulatory adjustments.

To develop my argument, I start by reviewing the technological characteristics of what is typically dubbed “machine intelligence” and compare it with our current understanding of human cognitive processes. Specifically, I will show how the current wave of machine intelligence is correlated with significant scale effects that make economic concentration a prerequisite for further technological development. Through an overview of empirical studies I will demonstrate the extent to which such concentration effects can already be observed and discuss the specific underlying mechanisms. Based on this analysis, I will argue that this tendency for economic concentration has some other, undesirable consequences from a macro-economic standpoint, including a slowdown in technological diffusion and a deceleration of productivity growth. My argument, therefore, consists in considering the current technological paradigm around AI as one of “supply push,” driven predominantly by technological considerations, rather than one of “demand pull,” oriented by policy goals regarding the development of productive forces and sustainable societies.

The way the AI trilemma is being developed in the following relies on an extensive review of the available evidence as it is being brought together by computer scientists, economists and policy experts to form a new, coherent understanding of the current difficulties that help understanding the apparent contradiction of a seemingly accelerating technological progress and a manifest difficulty to detect this progress in improvements in economic and social indicators.

### 2.2. The AI trilemma as a supply push paradigm

**Figure 4:**
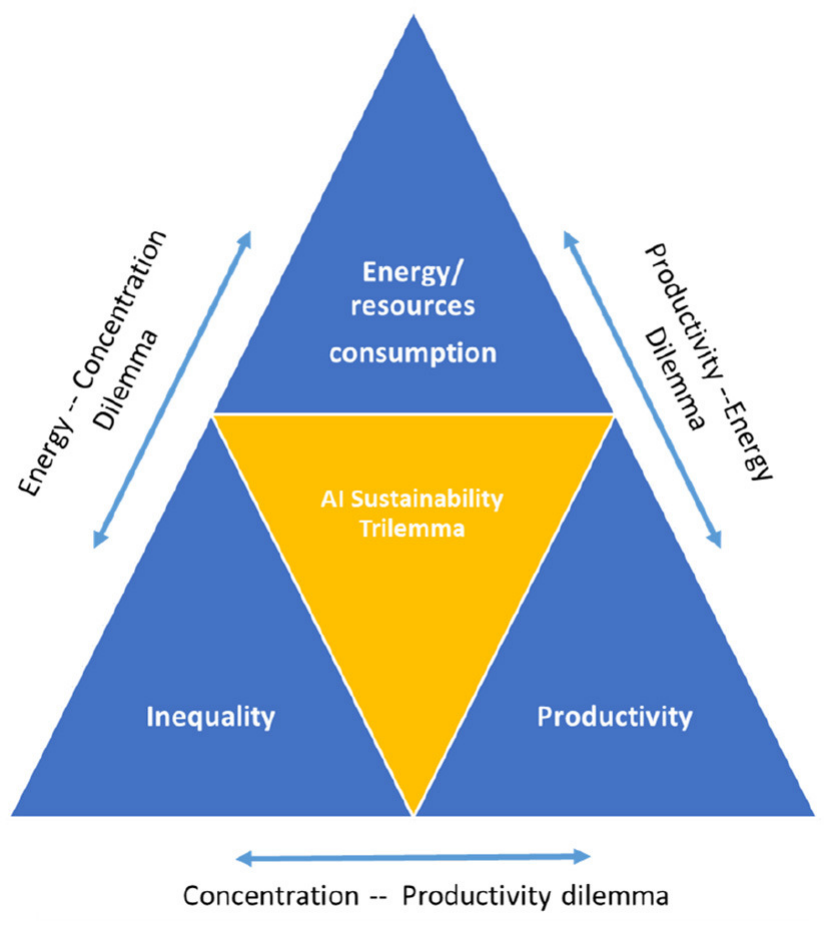
The AI sustainability trilemma in a nutshell.

Figure 4 summarizes the key message of the AI sustainability trilemma: We cannot have low inequality, high productivity and ecological sustainability simultaneously, at least not when pursuing the current technological paradigm underlying the development of AI-powered automated decision making systems. As such, the AI trilemma is composed of three, interrelated dilemmata of which only two can be solved simultaneously at the expense of the third one.

Specifically, the AI trilemma consists of the following three interrelated dilemmata:

- The productivity-energy dilemma (Figure 4, upper-right leg): Rising (labor) productivity can only be achieved through the replacement of human labor by machines at the expense of higher use of energy (electricity). This is not specific to the AI revolution. In the case of the digital economy, it implies that human cognitive work is being substituted by machine intelligence. In the next section we will see more closely that this often means that the energy efficiency of decision-making processes actually declines rather than improves. From the storage of data in cloud computing centers, to data analysis by high-performance computers, to the power consumption of even the smallest mobile digital devices needed to stay connected, the digital economy is already using up more than 6 percent of average electricity consumption. And the trend is accelerating. Without major efficiency gains, electricity consumption is expected to rise to over 20 percent in 2030 (Jones, 2018). This dilemma could only be overcome if productivity were to rise beyond what humans could achieve with the same amount of energy expended. As we argue, this is currently not the case.
- The energy-economic concentration dilemma (Figure 4, upper-left leg): For energy efficiency to increase rather than to fall, data concentration needs to grow further in order to exploit the variation of information in large samples. This is the logic currently underlying the development of approaches such as Large Language Models that exploit almost the entire (English-speaking) library. Given the network externalities involved in data collection (which we will discuss in more detail below), market concentration is bound to worsen, at least within the small segment of data collection and algorithm training. Such concentration of data collection can indeed enhance energy efficiency and hence yield productivity gains but only at the level of individual companies. At the aggregate level, this concentration worsens economic inequalities. This dilemma could only be overcome if access to data were regulated as a public good that allows strong competition among data users. In Section 4, we will discuss different options how this could be achieved.
- The concentration-productivity dilemma (Figure 4, bottom leg): Higher income inequality, especially in mature economies, is associated with lower productivity gains. As incomes are getting more concentrated at the top, aggregate demand grows more sluggishly, slowing down embodied technological change, i.e., that part of technological progress that requires investment in new machines. Whether higher productivity growth increases or declines inequality, on the other hand, depends on whether and how quickly new technologies diffuse throughout the economy. Highly specialized technologies that benefit only few sectors might permanently lift inequality when other sectors of the economy cannot from its advantages. In contrast, General Purpose Technologies are thought to “lift all boats,” albeit sometimes with a long delay, creating a J-curve effect (Brynjolfsson et al., 2021) with increases in unemployment in the short run and faster job growth in the long run (Chen and Semmler, 2018). In Section 4, we discuss possible ways of addressing the growth-depressing consequences of higher economic concentration.

There is indeed some debate regarding whether a J-curve effect is relevant in understanding why major economies have not yet seen productivity improvements commensurate of what has been expected from the latest wave of technological advancements. Depending on how flat the “J” is, the effect can take several decades, related to major sectoral restructuring and work-process re-organization. Ernst (2022) argues that because of the rise in inequality triggered by the specific conditions under which digital technologies evolve, it is rather unlikely to see a fast diffusion of these new applications spreading through the economy. In the worst case, these benefits might never materialize broadly. In other words, it is increasing market concentration of digital companies and widening income differentials that prevent stronger growth for all. Digital growth is not inclusive and—depending on the application—it is not resource efficient.

What explains this AI sustainability trilemma? This paper argues that the trilemma—low growth, greater inequality and high energy consumption despite rapid technological progress—is mainly due to the specific technological regime in which the digital economy currently operates: Under the current regime of intellectual property rights, energy efficiency of silicon-based information processing tools can only be achieved through high degrees of data concentration, preventing economy-wide productivity spillovers while generating significant economic inequalities. In other words, it is a supply-push technological paradigm driven by the specific conditions under which technological companies develop their applications. This "weightless economy" now occupies the largest place and leads to market distortions that have so far received insufficient attention (Haskel and Westlake, 2017). Moreover, AI-powered tools trigger various forms of inequality beyond the failure to diffuse its benefits more widely. Indeed, at the micro level, too, problems are emerging that perpetuate existing inequalities. The use of historical data, for instance, necessary to train AI routines, often reflects discrimination, specifically of women or ethnic minorities in the labor market. If an AI-routine is fed with such data without a corresponding filter, the disadvantages will be perpetuated, for instance through continued discrimination in hiring processes. Several major tech companies have already experienced this to their disadvantage. Taken together, the specific institutional and technological characteristics of artificial intelligence and AI-based innovations cause and perpetuate the AI sustainability trilemma. In order to offer possible ways out, however, we first need to better understand what is driving these three different elements of the trilemma in the next section.

## 3. Understanding the mechanisms of the trilemma

### 3.1. Why are brains so much more efficient than computers?

A core assertion of the AI trilemma is that computers are highly energy intensive. Therefore, their massive use in the current digital transformation of our economies comes at a significant cost for the environment, specifically in form of the use of electricity and its related carbon footprint. Looking at it from a total factor productivity perspective—i.e., considering all input factors, labor, capital and energy—we start by exploring the first axis of the AI trilemma: the trade-off between using computing power vs. brain power in the drive toward higher levels of productivity. This first section starts by looking into the reasons why digital tools in general—and machine learning in particular, at least as it is currently being conceived—are high consumers of energy. I discuss key differences between brains and computers, arguing that despite many broad similarities, their underlying architecture and information processes show remarkable differences that explain much of why brains are much more efficient than computers. I also discuss how recent changes in the way computer algorithms have evolved have integrated ideas inspired by neurological research, producing remarkable improvements in computing performance. My core argument in this section is that the way computers are currently being used is unsustainable from an ecological point of view. That is not to say that a different kind of use could not prove beneficial for society, but it would require reorienting our current technological paradigm away from trying to substitute for human cognition toward a paradigm where computers and brains are complements^3^.

Key for the argument in this section will be to understand the trade-offs involved between the functioning of a computer in comparison to the brain. This might come as a surprise for some as computers are often being seen and modeled following the architecture of the brain. Indeed, seeing the computer as the (better) version of the brain has a long history, going back to the early beginnings of the computer age (Cobb, 2020, ch. 12). Yet, there are fundamental differences in the working of a computer and a brain, beyond the physical characteristics of both (inorganic vs organic matter).

What adds to confounding both—computers and brains—is the fact that key components with similar function are present in both: Memory and circuits, i.e., structured connections between elementary units that can recall previously stored information—using transistors in the case of computers and neurons in the case of brains. Both elements have been shown to be essential for information processing. Indeed at a fundamental level, all mathematical functions can be represented by a suitable connection of basic logical gates, represented as neural networks, which makes the comparison of computers and brains particularly appealing (Hornik et al., 1989). Moreover, progress in computing performance over the past decades has been driven to a non-negligible part by improvements in algorithm design, often inspired by a better understanding of some of the key principles behind the workings of the brain. The exponential development and use of neural networks, for instance, was responsible for vast improvements over and above what simple hardware developments would have made possible (Sherry and Thompson, 2021).

As a consequence, many researchers consider that a convergence of computers toward brains is underway. Moreover, the rapid growth in applications around artificial intelligence suggests that computers would eventually not only work in a fashion similar to brains, they would even follow the same information process, making predictions based on limited information inputs (Friston, 2010; Agrawal et al., 2018). And yet, a direct comparison reveals significant differences in terms of performance and efficiency (see Table 1). In particular, a trade-off becomes apparent regarding the energy consumption and the precision/speed at which calculations are being carried out: individual human neurons are rather slow and imprecise when it comes to processing information. At the same time, they turn out to be much more powerful than transistors in computers, displaying much more complex patterns of activity than a simple binary activation potential (Gidon et al., 2020). On the other hand, computers can calculate at a significantly higher speed and precision, even though most of them dispose of less transistors and connections with much simpler activation patterns^4^. Moreover, this higher precision and speed comes at a significant price tag in the form of higher energy consumption.

**Table 1:** Comparing (traditional) computers and brains.

**Properties**	**Computer**	**Human brain**
Number of basic units	Up to 114 billion transistors	~100 billion neurons; ~100 trillion synapses
Speed of basic operation	20 teraflops/s.	<1,000/s
Precision	1 in 18.4 quintillion (for a 64-bit processor)	~1 in 100
Power consumption	up to 215 watt	~10 watt
Information processing mode	mostly serial with 20 cores	serial and massively parallel
Input/output for each unit	1–3	~1,000
Signaling mode	digital	digital and analog

Note: Based on an Apple M1 Ultra chip in 2022. Flops, floating-point operations per second.

Source: https://support.apple.com/en-am/HT213100 Herculano-Houzel (2009); Luo (2015).

Similarly, computers are significantly better at long-term storage of information (memory), which can span several decades, depending on the physical characteristics, the rate of technological obsolescence and processes to transfer information from one (digital) medium to another. In contrast, humans have difficulties in recollecting precisely even personal information, can easily be manipulated in what they remember (Shaw, 2016) and “suffer” systematically from forgetting due to the plasticity of the brain that adjusts to external input, something a computer cannot do (Ryan and Frankland, 2022).

Several architectural differences between computers and brains seem to explain a large part of the observed differences in performance, albeit computer scientists are keen in trying to close the gap regarding some of them. The question then becomes: if the architectural differences can be closed, would computers still perform better than brains where they currently have their comparative advantages? In other words: would it not be preferable to improve computers along the dimension where they currently have an advantage rather than trying to emulate the brain? At least from an economic point of view, such a trade-off would call for a more careful assessment of the use of digital tools depending on where their comparative advantages lie. In the following, I focus on four differences that are relevant from an efficiency point of view^5^.

A first difference, as noted in Table 1, stems from the parallel structure of the brain in comparison to the mostly serial way a computer functions. The massive expansion of machine learning approaches in computer science demonstrates that enormous efficiency gains can be achieved by parallelizing calculations in the computer. Essentially, neural networks that lie at the heart of recent progress in artificial intelligence use layers of parallel nodes stacked one upon each other, similar to the structure found in the brain, at least to a first order. Researchers increasingly recognize, however, that it is not only the parallel structure but also the specific way in which neurons are connected that explains performance differences (Luo, 2021). Indeed, the importance of a particular network topology in explaining this network's function is currently an active area of research and some of the insights are already being reflected in the way neural networks are being set up in order to further enhance their performance (Zambra et al., 2020). Related, the brain seems to be hardwired for particular tasks that are important for our social experience. For instance, our capacity to recognize faces (Alais et al., 2021) or letters (Turoman and Styles, 2017) seem to be hard-wired in our brains, whereas computers need to learn this. Similarly, we all seem to benefit from a universal grammar that allows us to learn language even without ever being exposed to the full richness of a language, a point made long ago by Noam Chomsky^6^. Such “pre-training,” although increasingly used in ML-applications makes our brain particularly energy-efficient if only less flexible.

A second difference lies with the particular way memory is structured in the brain. For one, memory loss as discussed before seems to play a significant role in enhancing a brain's energy efficiency by gradually removing information no longer needed (Li and van Rossum, 2020). Moreover, rather than having a fixed-size memory chip that stores all our information, memory is distributed and stored dynamically. Information, therefore, does not need to be shifted around and read out but is accessible exactly where it is needed. This has inspired recent research to develop integrated memory-computing circuits that allow information being stored where calculations are taken place, so called “mem-resistors” (Zahedinejad et al., 2022). So far, this remains experimental and has not yet been successfully implemented in large-scale computing but shows that significant efficiency gains even in hardware design are still available.

A third, and for our argument most decisive difference lies in the way information is being recorded in neurons in comparison to computer bytes. Indeed, computers process information in the form of small, fixed-sized chunks, so called bytes, in binary format. Regardless of the specific computer type, at any point in time during the operation, a significant number of the individual bits that compose each byte are active. In other words, computers use “dense representation” of information. More importantly, every time such a bit loses its action potential through a computing operation, energy is being released. In contrast, neurons have been shown to operate with sparse representations, where individual dendrites of a neuron are being activated when a certain (small) percentage of a large set of potential links is active, often less than 5 per cent (Ahmad and Scheinkman, 2016; Hawkins and Ahmad, 2016; Hole and Ahmad, 2021). Not only do operations on sparse representations use much less energy than those of dense ones—most operations involve zeros—they are also particularly robust against errors: Calculations by Hawkins and Ahmad (2016) demonstrate that for typical synapses error rates can reach 50 per cent without neurons losing their capacity to properly identifying underlying patterns. Such robustness against errors is an additional contributor for energy efficiency as it avoids costly error correction of calculations that need to be done on standard computing devices, in particular for critical hardware.

Finally, while these architectural differences primarily point to differences in the hardware, sparsity is also an important issue regarding algorithmic differences between computers and brains. As highlighted by Kahneman (2011), humans dispose of two main modes of decision making: slow, optimizing and calculating decision processes and fast, heuristic routines. The latter might come with cognitive biases but allow for quick decisions, in particular relevant in periods of stress and high threats. Heuristics are typically domain-specific, which is why their application to other domains induce cognitive biases by not considering all relevant options (Gigerenzer et al., 2011). At the same time, they are fast and energy-efficient. A role performed by the brain in this regard is to identify the specific situation and to mobilize the relevant resources for each decision problem. In contrast, algorithms currently employed in computers will systematically mobilize all available resources for any problem. Integrating these considerations there are shifts toward the use of more specialized CPUs that focus on particular tasks with more efficiency. So far, however, this more modular and specialized set-up has not reached the level of sophistication of the brain.

Taken together, the specific advantage of computers lies with fast, high precision calculations, such as those needed to design high-tech devices or to search quickly through the available library of human knowledge (or protein folding for that matter). In contrast, human brains have evolved to respond to particular challenges posed by our social environment in which empathy and understanding social settings play a fundamental role. Here, coordination, collaboration and adaptability to changing (social) circumstances are key for (collective) success, a task that is difficult for a computer to achieve as it is programmed for a (fixed) number of tasks. A first result of this comparison of the relative performance of computers vs brains, therefore, is the complementarity rather than substitutability of brain vs. computing power. This ties nicely with other research indicating the importance of AI as a transformative force rather than a disruptive one (Fossen and Sorgner, 2019; Carbonero et al., 2021). It also implies that current attempts to generate productivity gains by massively substituting labor for computers will not lead to the expected outcomes. Rather it will lead to a worsening of the energy bill of those companies that rely on such technologies.

As a consequence, technological developments of digital devices in general and AI-powered tools in particular suggest an exponential rise in the ecological footprint under the current technology paradigm (Jones, 2018; Thompson et al., 2020). A simple projection of the growth in model size that are driven by rising demands for precision shows that both the economic and ecological costs would quickly become unsustainable (see Table 2). As noted by the authors, this projection is a simple illustration and the trajectory unlikely to be followed literally as economic, financial and ecological constraints would prevent it from happen. One area, where this can already be observed regards applications around cryptocurrencies where several jurisdictions have issued restrictions or outright bans for so-called “mining” of currencies on their territory, mostly for reasons related to the rising energy costs (with knock-on effects on other activities in these countries).

**Table 2:** Computational costs of deep learning.

**Benchmark**	**Error rate**	**Polynomial**			**Exponential**		
		**Computations** **required**	**Environmental** **cost (*CO*_2_)**	**Economic cost** **($)**	**Computations** **required**	**Environmental** **cost (*CO*_2_)**	**Economic cost** **($)**
ImageNet	Today: 11.5%	10^14^	10^6^	10^8^	10^14^	10^6^	10^6^
	Target 1: 5%	10^19^	10^10^	10^11^	10^27^	10^19^	10^19^
	Target 2: 1%	10^28^	10^20^	10^20^	10^120^	10^112^	10^112^
MS COCO	Today: 46.7%	10^14^	10^6^	10^6^	10^15^	10^7^	10^7^
	Target 1: 30%	10^23^	10^14^	10^15^	10^29^	10^21^	10^21^
	Target 2: 10%	10^44^	10^36^	10^36^	10^107^	10^99^	10^99^
SQuAD 1.1	Today: 4.621%	10^13^	10^4^	10^5^	10^13^	10^5^	10^5^
	Target 1: 2%	10^15^	10^7^	10^7^	10^23^	10^15^	10^15^
	Target 2: 1%	10^18^	10^10^	10^10^	10^40^	10^32^	10^32^
CoLLN 2003	Today: 6.5%	10^13^	10^5^	10^5^	10^13^	10^5^	10^5^
	Target 1: 2%	10^43^	10^35^	10^35^	10^82^	10^73^	10^74^
	Target 2: 1%	10^61^	10^53^	10^53^	10^181^	10^173^	10^173^
WMT 2014 (EN-FR)	Today: 54.4%	10^12^	10^4^	10^4^	10^12^	10^4^	10^4^
	Target 1: 30%	10^23^	10^15^	10^15^	10^30^	10^22^	10^22^
	Target 2: 10%	10^43^	10^35^	10^35^	10^107^	10^99^	10^100^

Computations required in Gflops.

Source: Thompson et al. (2020), p. 14.

Regardless of the limits to growth for specific applications, a key challenge in promoting more efficient computing procedures and in assessing which tasks can better be carried out by humans rather than machines remains the proper assessment of the energy consumption involved over the entire computing value chain, from data collection, storage to machine learning and data use (García-Martín et al., 2019; Henderson et al., 2020).

### 3.2. Information rules: Consequences for market structure

A direct consequence of this high energy consumption is a rising market concentration among AI producing companies and a concentration of AI applications around the most promising—i.e., most profitable—applications as shown in Figure 2. One of the direct consequences of the rising economic costs implied by the exponential increase in energy consumption is a “narrowing of AI research” (Klinger et al., 2022). As highlighted by the authors, this narrowing of AI research is linked to a focus on data- and computational-intensive approaches around deep learning at the expense of other approaches in artificial intelligence that might be more easily accessible by smaller research outlets and academic researchers. Indeed, the ubiquitous availability of large, unstructured databases and the exponential fall in computing costs since the 1980s have contributed researchers to focus on a particular branch of AI development, namely statistical and machine learning at the expense of earlier attempts using symbolic AI to program expert systems which are potentially more easily accessible by a wider group of developers.

Related, narrowing AI research and rising ecological and economic cost lead to market concentration, both in the development and training of new (large) models (Bender et al., 2021) and in related digital applications such as blockchain applications in cryptocurrency markets, where similar tendencies to oligopolistic concentration can be observed (Arnosti and Weinberg, 2022). This should not come as a surprise as any market that requires large fixed investment to enter will show signs of concentration. This does not need to be a problem if alternative products and services are available that are (close) substitutes, a situation of monopolistic competition, which is at the heart of many models of economic growth (Aghion and Howitt, 1992). However, the narrowing of AI research suggests that the offer of such potential close substitutes is also declining, which would indeed lead to a concentration of the market as a whole. Indeed, the tendency of digital technologies to lead to superstar firms that dominate their market with knock-on effects on both down- and upstream market power is increasingly well documented (Coveri et al., 2021; Rikap, 2021).

But there is another force that pushes the data economy toward concentration: the network externalities of data collection (Jones and Tonetti, 2020). Indeed, individual data has three characteristics that distinguish it from standard goods and services: (1) its provision is (almost) costless and often done as a byproduct of other activities (such as purchasing a good online; Arrieta-Ibarra et al., 2018); (2) once provided it can be shared and re-used without costs; and (3) finally, its individual value is almost negligible other than in some extreme cases (e.g., rare diseases). Only as part of a larger database will individual data generate some economic value, for instance in order to determine customer profiles or applicants' characteristics (Varian, 2018). Such network externalities are known to lead to concentration effects as has become obvious with the rising share of only a small number of platform and social media providers on global stock exchanges.

In principle, concentration due to network externalities can be productivity enhancing, provided that the productivity gains generated from data concentration are being shared with platform users. This can happen, for instance when platforms are price-regulated, a principle that has been applied with previous network monopolies in telecommunication or electricity distribution. In the case of data monopolies, this is almost never possible as the use of many of these digital tools is not priced and users pay these services through alternative means, more difficult to regulate (e.g., exposure to commercials). Alternatively, stiff competition by alternative platform providers could help share these productivity gains more widely, but many of the incumbent platforms have grown so big that they either pre-emptively purchase potential competitors (e.g., Instagram in the case of Facebook) or use predatory pricing strategies against possible newcomers in order to limit their growth or reduce entry altogether (as in the case of Amazon, see Khan, 2017). As a consequence, productivity gains remain highly concentrated among a few, ever larger firms that see their evaluations sky-rocket. In contrast, the average company in OECD countries has barely experienced any (productivity) growth despite an ever larger investment in digital assets (Andrews et al., 2015; Haskel and Westlake, 2017). Indeed, a simple calculation can show that these gains represented in the form of rising stockmarket evaluations have macro-economic proportions: If the entire stockmarket value of the five largest digital companies were to be paid out as an indefinite annuity, US GDP would grow by almost 1.1 per cent, a significant improvement^7^.

Distributional aspects of the rising use of AI do not only appear at the macro-economic level, they also arise at the micro- and the meso-economic level. A direct consequence of the increased capacity of algorithms to treat large databases is the possibility for much refined pricing strategies, so-called individual pricing (or price discrimination). Such approaches redistribute welfare gains from consumers to producers, which can, under certain circumstances, be welfare-enhancing to the extent that they allow to increase the overall volume of production. Indeed, it can be shown that these circumstances arise fairly easily, which would argue for a more relaxed stance on such price discriminating strategies (Varian, 1985, 2010). On the other hand, research increasingly demonstrates that with the scaling of AI, these welfare-enhancing output expansion is exactly what is lacking: Instead, customer discrimination is being used to exclude certain socio-demographic categories from being served. This is particularly problematic in applications for human resources management, for instance, where automated hiring tools often seem to apply overly strict criteria for selection, thereby excluding large parts of the applicant pool (“hidden workers,” Fuller et al., 2021). Often, this is being discussed as algorithmic discrimination due to biases in historical databases upon which these algorithms are being trained. More profoundly, however, the reason for these welfare-reducing effects of AI in such cases lies in the legal prerogatives to prevent open discrimination, thereby setting incentives for firms to restrict services to certain groups only.

Recently the debate has started to focus on the distributional impact of algorithms at the meso-economic level, specifically on issues arising from algorithmic collusion (OECD, 2017; Calvano et al., 2020). In a traditional setting, pre-agreement is often necessary in a market with only few players in order to move from the welfare maximizing price level (the “Bertrand oligopoly”) to a profit-maximizing but welfare-reducing higher price level with lower output (the “Cournot oligopoly”). Anti-trust regulators, therefore, spend significant effort in documenting such written or oral commitments to compete on quantities rather than on prices. In a world where prices can be adjusted almost instantaneously and through algorithms, such agreements are no longer necessary: algorithms would learn from each others behavior and tacitly agree on profit-maximizing pricing strategies (Ezrachi and Stucke, 2020).

There is substantial disagreement, however, as to whether such tacit collusion has already been observed or could even become a serious threat not only to income distribution but to efficiency-gains to be obtained from AI (Dorner, 2021)^8^. Evidence is available primarily from online platforms, such as online drug sellers or airline ticket pricing (Brown and McKay, forthcoming) but also retail gasoline market where prices adjust frequently and increasingly through the use of algorithms (Assad et al., 2020). Whether markets are prone to algorithmic collusion might depend on the characteristics of the product or service sold, including the frequency of trades, the degree of transparency and the homogeneity of products, besides the availability of algorithms that could exploit such opportunities (Bernhardt and Dewenter, 2020). Regardless of how widespread the phenomenon is today, however, traditional anti-trust regulation will have difficulties to identify such cases, precisely because of their tacit nature. There is, therefore, a risk that scaling up the use of AI in determining prices (and wages) will not only lead to further concentration and rent seeking behavior, it will also significantly reduce efficiency regardless of any labor displacement effects these technologies might have. Some options exist to regulate firm behavior through appropriate setting of fines and divestitures but current examples involving social media platforms suggest that such regulatory activism is likely met with strong resistance (Beneke and Mackenrodt, 2021).

### 3.3. Why do we not see more productivity growth?

The last aspect of our AI trilemma looks at the low and declining productivity growth observed in most advanced countries and major emerging economies. As noted above, economists have long noted a productivity puzzle between the apparent acceleration in technological progress, specifically around digital technologies, and the lack of observed productivity gains, at least at the national level (Brynjolfsson et al., 2019). To understand this puzzle, national productivity growth needs to be broken down into its components: Indeed, aggregate increases in productivity are the product of productivity improvements at the firm or factory level and the spread of these gains across the economy. Simply put, productivity = innovation times diffusion. The question therefore becomes twofold: Is the lack of observed productivity gains due to a failure of the digital economy and AI to push productivity at the individual firm level or is it related to a failure of such gains to diffuse through the economy more broadly. The answer researchers have given so far is: problems reside at both ends and are possibly linked.

At the firm level, the introduction of new technologies in general and AI in particular has always been confronted with a necessary re-organization of work processes (Dhondt et al., 2021). As such re-organization takes time and energy, a J-curve effect arises: Each new technology requires upfront costs in the form of restructuring that might actually depress productivity and firm profitability. Once these adjustments have successfully taken place, however, productivity will rise above the level at the start of the adjustment process (Brynjolfsson et al., 2021). At the firm level, evidence is indeed emerging that the recent surge in patenting around artificial intelligence and robotisation has led to a global increase in firm level productivity, especially among SMEs and in services (Damioli et al., 2021). Research specifically for the United States seems to suggest, however, that effects of AI are particularly strong in large firms that patent significantly (Alderucci et al., 2020). Looking at productivity spillovers, on the other hand, Venturini (2022) suggests that at least during the early periods of the transition toward automation based on AI and robotics, significant spillovers might have contributed to the observed productivity increases. In other words, despite increases in productivity at both the firm and the sectoral level that were driven by AI and robotization, aggregate apparent labor productivity growth decelerated, suggesting that other factors must have been holding back the possible positive contribution of AI on growth.

One possible factor might lie in the restructuring of production chains. Indeed, as highlighted by McNerney et al. (2022), as economies mature, production chains normally become longer, which increases their capacity to generate aggregate productivity growth from individual, firm-level or sectoral improvements in productivity. However, over the last 15 years, global trade growth has stalled, suggesting at least a stagnation if not shrinking of the length of production chains, which would suggest a loss in the capacity of AI to generate productivity growth at the aggregate level. Unfortunately, the evidence in McNerney et al. (2022) stops in 2009 but suggests that some of these dampening effects of slow global trade growth might indeed have started to appear toward the end of their observation period.

Closer to the argument developed here, the narrowing of AI research suggests another possibility, following Zuboff (2019): Indeed, the rapid increase in AI applications might be concentrated around surveillance software and human resources management tools that impact workplace organization more than it contributes to overall productivity increases. Part of the restructuring induced by such software impacts not so much the overall innovative capacity of firms but rather the type of innovation carried out, with little impact on firm profitability and employee output. In other words, rising investment in this type of AI focused on HR management helps more with overall information processing and incentive provisions than it does for value creation, which is why firm level studies suggests that only some firms seem to benefit from these tools.

At the macro level, another factor limiting aggregate productivity gains from AI is explored by Gries and Naudé (2020) expanding on Acemoglu and Restrepo (2019) and analyzing an endogenous growth model. The authors analyse the impact of AI-induced automation of tasks rather than entire jobs, demonstrating that regardless of the elasticity of substitution between AI and human labor, the aggregate labor income share falls, with adverse consequences for aggregate demand and productivity growth. When the elasticity of substitution is high, the displacement effect is always greater than the reinstatement effect of new tasks (Acemoglu and Restrepo, 2019). However, Gries and Naudé (2020) show that even in the case when the elasticity of substitution is low, the reinstatement effect fails to compensate for labor displacement in an endogenous growth setting provided that the benefits from AI are heavily concentrated among capital owners, a direct consequence from the distributional aspects of AI discussed in the previous section. In contrast to previous waves of automation, therefore, the data economy generates highly concentrated benefits that do not generate enough demand spillovers to push up growth on a broad basis.

A last factor, intimately related to the distributional consequences of the data economy concerns its impact on the degree of market competition, a point stressed by Aghion et al. (2021). Indeed, Schumpeterian rents arising from innovation such as AI need to be gradually eroded through the entry of new producers of highly substitutable goods and services in order to allow for a wide diffusion of productivity gains. This is the essence of Aghion and Howitt (1992)'s original work on creative destruction and subsequent empirical evidence. As demonstrated by Hidalgo and Hausmann (2009) and Pinheiro et al. (2021) when such growth models are prevalent in a large range of unrelated sectors they lead countries on a path of high and persistent economic development. In this case, monopolistic competition coupled with creative destruction ensures the continued upgrading of productivity across a broad range of sectors, a model that was followed broadly during the first two waves of industrial revolutions. However, with the arrival of digital capitalism and data markets, the data rents generated by platform providers and AI innovators only partly diffuse through the economy, thereby lowering labor income shares and aggregate demand, a trend observed since the arrival of the computing revolution in the 1980s that continues until today.

This ties well with another observation that has puzzled economists for some time: The decline in business creation and start-up activity over the past two decades (Bessen, 2022). Indeed, the trend toward rising market power across the globe is well documented, following directly from a lack of market contestability by smaller, younger firms (Eeckhout, 2021). As Bessen (2020) demonstrates, this trend toward industry concentration can be directly linked to the rise in the data economy and the related growth in proprietary information technology. Such industry concentration, even if driven by innovative products and services, are not without adverse consequences for aggregate productivity growth (De Loecker et al., 2020).

This then closes the loop of the AI trilemma. Despite the potential of creating substantial productivity gains at the firm level and some evidence for productivity spillovers, the potential for a broad-based increase in aggregate productivity is limited by the adverse distributional consequences of the way the data economy functions. Empirically, this shows up in a widening productivity gap between frontier firms and the rest (Andrews et al., 2015). At the same time, the high energy consumption not only limits the societal benefits of this technology; it is itself partly responsible for the high concentration of AI providers and a narrowing of AI applications. In this regard, the suggestions put forward by some observers to alter the regulatory environment of the data economy, for instance by modifying current regulation on intellectual property rights might not be sufficient to address the trilemma as presented here (e.g., Karakilic, 2019). We will see in the next section that solving the AI trilemma requires a more encompassing approach that targets the specific benefits that a widespread adoption of AI can have by mitigating its adverse ecological and social costs.

## 4. Solving the AI trilemma

Dissecting the underpinnings of the AI trilemma allows an understanding of how to address it. Key to any policy or regulatory intervention is that the trilemma is specific to the current technological paradigm under which the digital economy develops, not an inherent characteristic of the technology. Such paradigms are subject not only to the physical characteristics of a specific technology but also to the institutional framework under which the technology is being developed (Dosi, 1982; Bassanini and Ernst, 2002; Nightingale et al., 2008). Specifically, as argued in the previous section, the current technological paradigm is one of a supply-push, where technology develops mostly through individual company strategies. In this section, I argue that to overcome the AI trilemma a switch to a demand-pull technological regime is necessary where technology develops through a deliberate shift in the institutional framework geared toward applications beneficial from a societal perspective.

In the following, I offer three approaches to address the AI trilemma, each one targeting one specific axis of the trilemma as highlighted by Figure 4. What follows from the discussion in the previous section is that breaking the trilemma requires one of three things: an orientation of technological development toward complementary, efficiency enhancing innovations; a more equitable distribution of innovation rents; or a more widespread diffusion of productivity gains through restoration of competitive markets.

**Figure 5:**
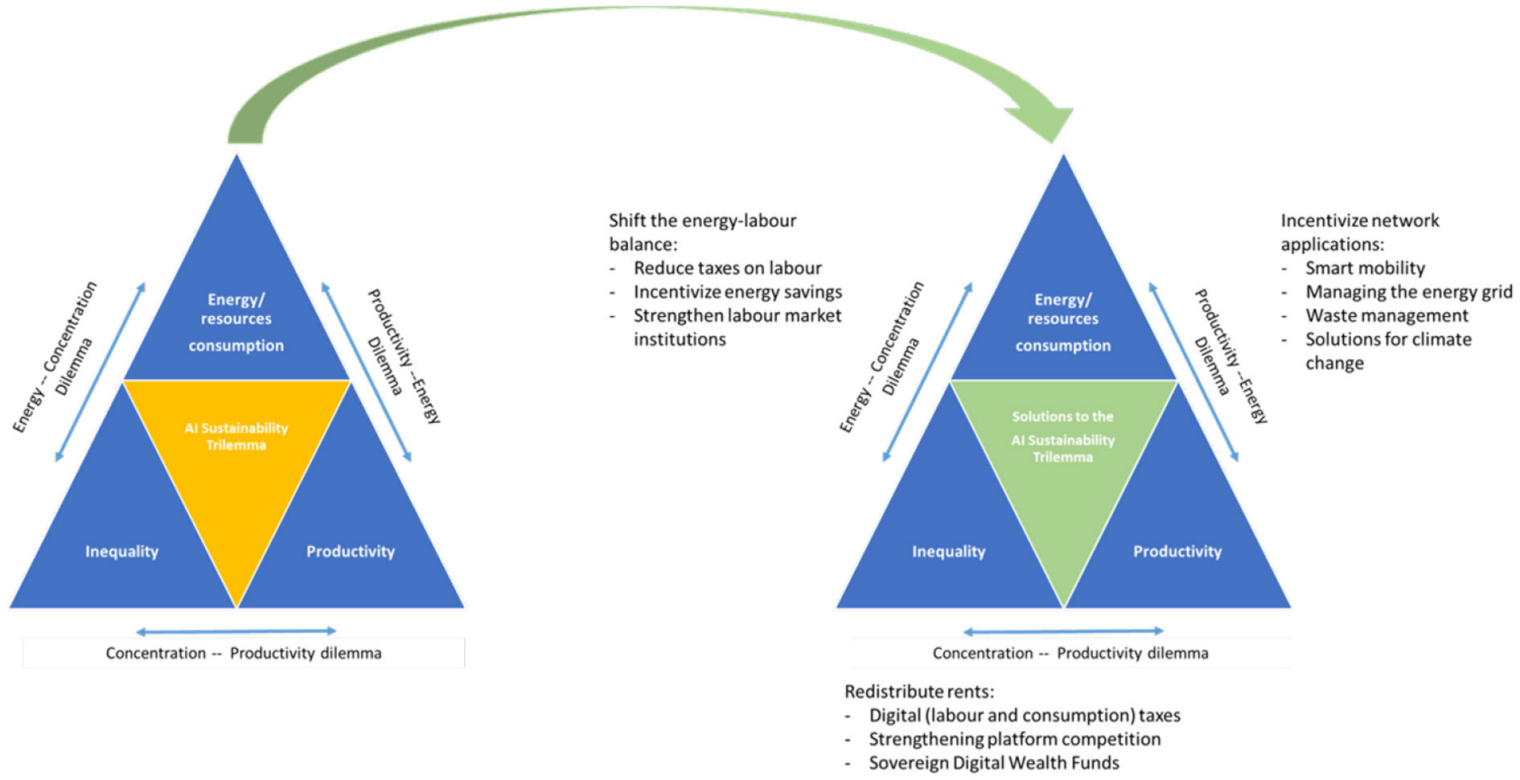
Solving the AI trilemma.

A first approach uses standard public economics: If the current technological regime under which AI development operates produces externalities (environmental, social, etc.), these need to be internalized through regulatory or institutional changes, for instance through changes in the corporate tax code or by strengthening labor market institutions. A second approach considers direct interventions to orient technological development through policy action into applications with high societal value that can lift productivity growth sufficiently to justify the additional energy consumption, i.e., an approach that will lead to an overall reduction in total resource consumption. A final approach focuses on the concentration dilemma, addressing the public goods problem of the current regime of digital technologies. The following Figure 5 summarizes the solutions for solving the AI trilemma that are being discussed in the following.

### 4.1. Solving the energy-concentration dilemma: Shifting the energy-labor balance

Addressing the AI trilemma faces two interconnected challenges: (i) steering technological progress into a direction that is at least neutral and ideally complementary to jobs (Mazzucato, 2021) so that the introduction of new machines strengthens the demand for labor; and (ii) ensuring that technological progress in general—and the increasing use of AI in particular—reduces its ecological footprint rather than to increase it (Acemoglu et al., 2012). However, as discussed in much of the literature on environmental transition, these two objectives often conflict, not least because the investment in new, environmental technologies requires time to allow for resources to be fully re-allocated. Moreover, many jobs in industries that have a heavy ecological footprint are often well-paying jobs for workers with less than graduate degrees (Montt et al., 2018). In other words, our AI trilemma induces a policy trade-off between better jobs and more energy efficiency, with both transitions possibly coming at the cost of a—at least—temporary slow-down or even reduction in productivity growth.

Here we want to suggest an alternative adjustment path that can tackle these problems directly and still solve the AI trilemma. This is made possible by the particular characteristics of AI, which did not exist to the same extent with previous forms of technological change, including technologies such as robots. For this, we need to extend our view on aggregate production to not only include energy but also organizational capital broadly understood:


Y=A(K,L,E,O)

where input factors are noted as *K* for capital, *L* for labor, *E* for energy, and *O* for organizational capital. Such extensions have a long history in economics, especially in firm-level empirical analysis (see, for instance, Atkeson and Kehoe, 2005). At the macro-economic level, however, improvements in organizational capital, *O*, are typically subsumed under the heading of “total factor productivity,” without clarifying whether these occur at the micro-, meso- or macro-economic level.

Current conceptualisations focus on AI as a technology that either replaces or complements jobs similar to previous waves of automation (Fossen and Sorgner, 2019). Notwithstanding the fact that economic analysis has increasingly focused on the impact of technology not on the individual job but on the underlying tasks that are being performed by a job (Autor et al., 2003, 2006; Autor, 2013; Acemoglu and Restrepo, 2019), AI is not considered to be distinct from previous forms of technological progress in this respect. However, as discussed in the opening part of the previous section, one specificity of AI is its capacity to process information in order to make predictions, for instance regarding the dynamics of a particular system. At the micro-economic level, such predictions can help an individual worker, for instance, in respecting a certain order in which to process the workflow by giving recommendations about the next step. Similarly, in a research environment, AI has been used to facilitate the discovery process of new drugs, thereby improving the productivity of the innovation process. At the sectoral level, AI can and has been used for dynamic pricing purposes (Calvano et al., 2020). Both can be thought of being complementary to labor, in the sense of a traditional production function. At the macro-economic level, these considerations add a new dimension. Here, applications exist that are not readily interpretable as either complements or substitutes for labor. For instance, AI tools are increasingly being used to improve the management of waste and electricity networks or help with improving the use and utilization of transport systems, including through inter-modal connectivity (see also the discussion in the next sub-section). None of these activities are directly linked to human labor (unless, for instance, one considers the commute to and from work as part of the aggregate production function, which typically it is not). Most of these applications of AI would, therefore fall into the category of innovations to improve total factor productivity.

Such innovations focused at improving resource efficiency are unlikely to have any direct employment effects but might impact comparative advantages of different sectors as they impact the way capital and labor is being used. Applications to improve waste management (e.g., in Barcelona), to help municipal officials to identify more rapidly infrastructure shortcomings (e.g., Amsterdam) or to improve the management of traffic systems (e.g., Delhi, Kuala Lumpur) reduces overhead costs. As such, they do not substitute for any current or future jobs (other than the engineers developing the software). However, to the extent that applications help to improve resources efficiency in particular industries or sectors, with effects on the comparative advantages of this industry both domestically and internationally, resources will be reallocated across sectors with implications for jobs and growth (Rentsch and Brinksmeier, 2015). Similarly, to the extent that cities benefit from AI differently, more advanced municipalities are likely to attract new businesses and jobs, leading to a geographical reallocation of resources. For the moment and to our knowledge, however, there is no good empirical understanding of the extent to which AI can help in improving resource efficiency in the aggregate, at the sectoral level or spatially, which precludes a proper quantitative assessment of this particular dimension of improvements in AI.

Such indirect effects of efficiency improvements on labor markets can be complemented by specific interventions that help strengthening labor to be complementary rather than a substitute. In particular, there are three areas where policy makers and social partners alike can help to steer technological change to become complementary to workers rather than substitutes:

- A first and most direct way of intervening to prevent excessive automation is *via* R&D incentives and tax credits: As highlighted in Figure 2, investment in AI is highly concentrated among a few areas, mostly associated with excessive automation (Acemoglu and Restrepo, 2019, 2020). Such interventions are always possible and might bring about a more balanced developed as regards the evolution of AI and its social impact. However, from the discussion of the AI trilemma, it follows that a broad-based support of advances in AI that are complementary to labor might not necessarily solve the energy problem at the same time. Rather, as with previous waves of technological progress, automation can come at the cost of excessive use of energy. In other words, direct interventions for AI development need to focus simultaneously on their resource-efficiency and labor-complementarity aspect in order to be effective when trying to address the AI trilemma.
- A second intervention works through reducing the tax burden on labor that has specifically in the US led to strong incentives for automation (Acemoglu et al., 2020). Instead, a shift of the tax burden away from labor toward energy consumption can address both the adverse resource and labor impact of AI. Indeed, as discussed by Ciminelli et al. (2019) an often overlooked channel of a revenue-neutral tax reform toward consumption taxes is that it strengthen labor supply incentives at the lower end of the income distribution, thereby partly correcting for its regressive income effect.
- Finally, the most indirect and challenging way to steer the degree to which a resource-efficient evolution of AI can produce positive outcomes on jobs and working conditions is by strengthening labor market institutions, such as work's councils that influence technological choices at the firm level (El-Ganainy et al., 2021). Such institutional arrangements have been shown to affect the way in which technologies are being applied and implemented at the workplace level. In the scenario envisaged here, activities would develop in sectors and occupations that would benefit from both AI-triggered resource efficiency improvements and institutional comparative advantages in favor of cooperative labor relations (Ernst, 2005).

A first approach to address the AI trilemma, therefore, lies with the necessity to steer AI developments in the direction of improving total factor productivity as an aspect for which AI is particularly suited and where its potential to substitute for labor is minimized, simply because so far none of these network functions are fulfilled by human labor. Complementary interventions are needed, however, to address possible adverse effects of resource-efficiency enhancing AI applications in labor-intensive occupations and sectors. In the following, we discuss how the particular network complementarities implied by AI might challenge such an approach.

### 4.2. Solving the productivity-energy dilemma: Incentivize the use of network applications

Not all AI applications are affected to the same extent by the AI-trilemma. Especially the already mentioned network applications have the potential to perform particularly well when it comes to lower resource consumption and improve inclusivity. Well-trained AI routines, for example regarding electricity management or water consumption in agriculture already reduce the burden on the environment today and offer possibilities to address climate change effectively (see, for instance, Rolnick et al., 2023). Digital technologies are likely to play a key role in helping our societies to adapt to rising climate risks by making critical infrastructure more resilient (Argyroudis et al., 2022). Furthermore, such solutions also offer opportunities for cost-effective knowledge transfer to developing countries, where there is still a great need to catch up on modern technologies adapted to local conditions. Companies such as Google and Microsoft have already discovered this need and have begun to establish their own research centers in some developing countries. And local solutions, especially in agriculture, also show potential productivity gains in these countries (Ernst et al., 2019). In the following, we briefly discuss three areas where the network management of AI tools can prove of particular support: energy management, traffic management and remote work.

Energy management is particularly high on the agenda for AI applications. Managing complex electricity grids across different jurisdictions (particularly acute in Europe) and diverse energy sources as energy production is increasingly ensured by renewables pose formidable challenges to grid management. Failure for proper management and anticipation of external (weather) events can lead to grid outage, as experienced in Texas during the winter of 2020/21, for instance. Combining Internet of Things devices and smart meters into smart grids has been a focus of development in the energy industry (Ahmad et al., 2022). Beyond grid management, preventive maintenance and smart consumption are also major areas of research and development that can help both in reducing risks of outage and overall consumption^9^. Power consumption management, in particular, has become an active area of research for tech companies in their attempt to reduce their own carbon footprint and is likely to contribute to a substantial reduction of the energy-intensity of AI models^10^.

Mobility management as part of a smart city policy is another area of high potential for digital tools to address the AI trilemma. Logistics management is an area where modern communication networks and complex supply chain management is already making use of AI-powered tools^11^. Similarly, applications regarding modal interconnectivity for individual transportation receive increasing attention, especially in areas where transport supply elasticity is limited. These applications are meant to facilitate personal traffic in dense urban settings that provide alternative modes of transportation for the same route. Managing such traffic networks through AI-powered tools will allow to improve traffic fluidity and manage limited infrastructure capacity more effectively (Nepelski, 2021).

A final area to be considered here is the role AI can play in our current transition to a higher share of remote work. Advanced economies, in particular, have demonstrated surprising resilience with respect to requirements to work from home that came with the pandemic-induced lockdowns in 2020/21. Dubbed “potential capital,” the large share of digital infrastructure and personal computing devices allowed a large part of the workforce to continue their economic activities and limit the economic outfall of the health crisis (Eberly et al., 2021). As economies are recovering from this shock, remote work will remain a reality at least for part of the workforce, creating challenges in terms of scheduling, information sharing and networking (Kahn, 2022). In particular the development and maintenance of personal and professional ties that are important for economic advancement have been shown to be critically affected by remote work (Yang et al., 2022). So far, gains from going remote have been meager. Both business leaders and employees are still trying to figure out how best to make use of the new flexibility that working from home offers (Cappelli, 2021). Here again, AI tools can prove an important answer to solve this challenge at least partially, developing complex scheduling software and helping to maintain information integrity across highly distributed networks of employees.

Taken together such applications make use of the potential of AI tools to directly address questions of aggregate resource efficiency rather than substituting capital for labor, thereby bringing us closer to resolving the AI-trilemma.

### 4.3. Solving the concentration-productivity dilemma: Redistribute rents

As the previous discussion makes clear, these changes require adjustments not only in the way technology is being developed but also in the institutional and policy settings under which innovators and businesses operate. In concluding this section, three approaches are being discussed that have the potential to address both the technological and the distributional aspects of the AI trilemma:

A first, traditional answer is to try to use taxes to better capture capital gains, while at the same time shifting the tax pressure from labor back toward capital. This has often been discussed in connection with a robot tax (Merola, 2022). On the one hand, it would allow the enormous profits of digital companies to be captured. On the other hand, tax fairness would be restored, which could relieve the factor labor and ease the pressure toward rationalization and job losses. However, in a global economy, governments have tight limits on how much they can tax internationally operating companies. Attempts to extend taxation to the consumption of digital services instead of profits are being resisted by those countries that are home to a myriad of large, digital companies. Moreover, as mentionned before, the tax burden needs to shift away from labor and toward energy consumption if the trilemma is to be properly addressed.

A second, more innovative approach is to ensure greater competition between digital enterprises, for instance by making it easy to transfer data between platforms using uniform standards and protocols. Some solutions also propose data ownership in order to provide a monetary incentive for those who make their data available by using the platforms. So far, however, none of these solutions are fully developed and practicable yet. Moreover, only very few users can derive relatively large profits from such approaches, while the vast majority of them would have little to expect. The incentive to switch platforms or to reap monetary rewards would be too low to solve the AI trilemma.

A final, little debated solution is to set up a sovereign digital wealth fund that participates widely in the digital economy. Currently, sovereign wealth funds (SWF) have been set up in relation with tangible public goods such as natural resources. Leaving the exploitation of such resources to private companies, sovereign wealth funds invest in these activities to the benefits of a public shareholder, such as the government. This allows the benefits of such public goods to be passed on to a broad group of people. However, instead of feeding off oil wells (as in the case of Saudi Arabia, Norway) or fish stocks (as in Alaska), a Sovereign Digital Wealth Fund would be financed by taxes and new debt, in order to generate returns by investing in a broad fund of innovative digital companies. At the same time, such a fund, provided it invests deeply enough, would also be able to directly influence the operative business in market-dominant companies in order to prevent the exploitation of such positions. Similarly, the fund could also aim at exerting influence at the micro level to ensure that ethical and ecological standards are met when using AI. Existing SWFs have increasingly invested in technology sectors, without, however, taking an active stance as regards the technological development nor the economic impact of the companies they have invested in Engel et al. (2020).

None of the solutions outlined here will be sufficient in themselves to resolve the AI trilemma. National solutions often do not provide sufficient guarantee that all market participants will actually be offered the same conditions. International approaches, especially in the area of taxation, are slowly gaining acceptance, but often only at the lowest common denominator. Innovative solutions such as data ownership require institutional changes, which will most likely take some time to be established and enforced. However, an approach that addresses all three proposed solutions should make it possible to find initial answers to the AI trilemma while at the same time offering new, individualized proposals that optimize the potential that AI holds for jobs, income and inclusiveness. The future of work demands not only technological innovations, but also political and institutional ones.

## 5. Conclusion

The article introduces and discusses the AI sustainability trilemma, the impossibility to achieve ecological sustainability, (income) equality and productivity growth under the current technological paradigm. It presents arguments as to why the energy-intensive nature of current computing capabilities combined with strong network externalities leads to market concentration, narrow AI research and weak (aggregate) productivity gains. The paper also discusses possible answers to this trilemma, demonstrating the potential for directed technological change toward network applications, for instance in electricity and mobility management, as a way to improve total factor productivity that will lead to a lower overall ecological footprint and higher aggregate productivity without worsening inequality. Such directed technological change requires, however, both technological and institutional changes to take place in order to reduce the tendancy of the digital economy toward market concentration.

Much of the potential to overcome the AI trilemma remains speculative at this stage, simply because the overall impact of directed technological change has not been tested or implemented at scale. Some of the institutional shifts required are likely to be resisted by strong incumbents that might lose their market dominant positions. At the technological level, individual applications show the potential to address the shortcomings of the current direction of technological change but real-world examples are lacking at the time of writing of this article. As new applications are being developed and implemented at scale, careful empirical research is necessary to assess the extent to which they can truly address the AI trilemma and possible additional policy changes required to fully benefit from the technological evolution around digital tools and artificial intelligence. Policy shifts that encourage less resource use and reduces (tax) penalties on hiring labor can help induce the development of more socially beneficial digital tools. A more active stance, for instance, *via* the establishment of Sovereign Digital Wealth Funds similar to existing models on natural resources management should be used to accelerate the transition toward a new technological paradigm that overcomes the AI trilemma. The switch from a supply-push to a demand-pull technological regime as argued for in this paper requires further analysis regarding the specific applications that can help overcome the trilemma. In particular, beyond the technological feasibility of these changes, the specific political and institutional roadblocks need to be carefully identified and addressed, opening yet another interesting research avenue.

## Data availability statement

The original contributions presented in the study are included in the article/supplementary material, further inquiries can be directed to the corresponding author.

## Author contributions

The author confirms being the sole contributor of this work and has approved it for publication.

## Conflict of interest

The author declares that the research was conducted in the absence of any commercial or financial relationships that could be construed as a potential conflict of interest.

## Publisher's note

All claims expressed in this article are solely those of the authors and do not necessarily represent those of their affiliated organizations, or those of the publisher, the editors and the reviewers. Any product that may be evaluated in this article, or claim that may be made by its manufacturer, is not guaranteed or endorsed by the publisher.

## Footnotes

^1^Cryptocurrencies and blockchain applications more broadly are not strictly relying on artificial intelligence. However, many of their applications do, including latest developments around Decentralized Autonomous Organization applications (DAO) that execute certain functions autonomously or market trading applications to anticipate price movements in these currencies. Many of the ecological implications discussed in this article relative to AI applications do carry over to other digital tools such as the use of blockchains.

^2^Foundation models, a term first coined by the Stanford Institute for Human-Centered Artificial Intelligence, are generic models trained on a large set of unlabeled data that can be re-purposed for a specific set of tasks. For example in natural language processing, the Bidirectional Encoder Representations from Transformers (BERT) model has been trained on a large corpus of the English language; the model can then be refined for specific tasks to recognize English sentences in technical applications, such as to identify the similarity in the description of skills in different classification systems (see, for instance, Fossen et al., 2022).

^3^My argument is different from a general backlash against technological progress and rather stresses the comparative advantages each cognitive technology brings, see, for instance, the criticism expressed here: https://datainnovation.org/2022/01/innovation-wars-episode-ai-the-techlash-strikes-back/.

^4^The hardware evolution continues to add significant amounts of transistors every year. At the time of writing, the largest computer, the Chinese-built supercomputer Sunway TaihuLight counted around 400 trillion transistors across all its CPUs. https://en.wikipedia.org/wiki/Transistor_count.

^5^There are further differences that are less relevant for our argument, such as embodiment. A good overview of the differences between the brain and how artificial intelligence is being set up, see https://www.technologyreview.com/2021/03/03/1020247/artificial-intelligence-brain-neuroscience-jeff-hawkins/.

^6^https://thebrain.mcgill.ca/flash/capsules/outil_rouge06.html, https://theconversation.com/our-ability-to-recognise-letters-could-be-hard-wired-into-our-brains-83991

^7^At the end of 2021, the fifth largest digital companies were (by stockmarket valuation): Apple ($2.91 T), Microsoft ($2.53 T), Alphabet/Google ($1.92 T), Amazon ($1.69 T), and Tesla ($1.06 T). Assuming an annuity with an infinite time horizon paid out at the historical average real return for US treasury bonds (around 2.47 per cent p.a.), this would lead to a total annual pay-out of around $250 B or slightly less than 1.1 per cent of US GDP in 2021 ($23 T). Stockmarket valuations are taken from https://companiesmarketcap.com/, US GDP comes from the Bureau of Economic Analysis: https://www.bea.gov/news/2022/gross-domestic-product-fourth-quarter-and-year-2021-second-estimate and historical (real) treasury bond rates have been calculated on the basis of Jordà et al. (2019).

^8^See also https://www.autoritedelaconcurrence.fr/sites/default/files/algorithms-and-competition.pdf.

^9^ https://www.xcubelabs.com/blog/applications-of-ai-in-the-energy-sector/

^10^ https://ai.googleblog.com/2022/02/good-news-about-carbon-footprint-of.html

^11^ https://www.technologyreview.com/2021/10/20/1037636/decarbonizing-industries-with-connectivity-and-5g/?mc_cid=98f3a8206d&mc_eid=59ed455432
